# Barriers to pregnancy prevention for adolescents in rural Haiti: perceptions of healthcare providers

**DOI:** 10.1186/s12905-024-03136-6

**Published:** 2024-05-22

**Authors:** Shelbie Wooten, Emily Hurley, Nikolaus Schuetz, Melissa K. Miller, Jonathan Rodean, Emily Rupe, Kemi Lewis, Marie Daphnée Boncoeur, Abbey R. Masonbrink

**Affiliations:** 1 Pediatric Hospital Medicine, Children’s Mercy Kansas City, 2401 Gilham Rd, Kansas City, MO 64108 USA; 2 Department of Pediatrics, University of Missouri-Kansas City School of Medicine, Kansas City, MO USA; 3 Children’s Hospital Association, Lenexa, KS USA; 4 Division of Health Services and Outcomes Research, Children’s Mercy Kansas City, Kansas City, MO USA; 5 Department of Population Health, Medical Center, University of Kansas, Kansas City, MO USA; 6 University of Kansas School of Medicine, Wichita, KS USA; 7 Global Birthing Home Foundation, Leawood, KS USA

**Keywords:** Contraception, Pregnancy prevention, Haiti, Adolescents, Healthcare providers

## Abstract

**Background:**

Little is known about healthcare providers’ (HCPs) contraceptive views for adolescents in Haiti, who experience high rates of unintended pregnancy. We sought to describe HCPs’ perspectives on barriers and facilitators to contraceptive care delivery in rural Haiti.

**Methods:**

We conducted a cross-sectional survey and qualitative interviews with HCPs in two rural communities in Haiti from 08/2021–03/2022. We assessed demographics, clinical practice behaviors and explored contraception perspectives according to Theory of Planned Behavior constructs: attitudes, subjective norms, and perceived behavioral control (e.g., people’s perceptions of their ability to perform a given behavior, barriers and facilitators of a behavior).^15–17^ We used descriptive statistics to report proportions and responses to Likert scale and multiple-choice questions. Guided by content analysis, we analyzed interview transcripts through thematic inductive coding and team debriefing.

**Results:**

Among 58 respondents, 90% (*n* = 52) were female and 53% (*n* = 31) were nurses. Most reported always (*n* = 16, 28%) or very often (*n* = 21, 36%) obtaining a sexual history for adolescents. A majority agreed/strongly agreed that clinicians should discuss pregnancy prevention (*n* = 45, 78%), high-risk sexual behaviors (*n* = 40, 69%), and should prescribe contraception (*n* = 41, 71%) to adolescents. The most frequently cited provider-level barriers (i.e., significant or somewhat of a barrier) included insufficient contraception knowledge (*n* = 44, 77%) and time (*n* = 37, 64%). HCPs were concerned about barriers at the patient-level (e.g. adolescents’ fear of parental notification [*n* = 37, 64%], adolescents will give inaccurate information about sexual behaviors [*n* = 25, 43%]) and system-level (e.g. resistance to providing care from administration [*n* = 33, 57%]). In interviews (*n* = 17), HCPs generally supported contraception care for adolescents. Many HCPs echoed our quantitative findings on concerns about privacy and confidentiality. HCPs reported concerns about lack of contraception education leading to misconceptions, and community and parental judgement. HCPs expressed interest in further contraception training and resources and noted the importance of providing youth-friendly contraceptive care.

**Conclusions:**

While HCPs support contraceptive care, we identified actionable barriers to improve care for adolescents in rural Haiti. Future efforts should include increasing HCP knowledge and training, community and parent coalition building to increase contraception support and offering youth-friendly contraceptive care to offset risk for related adverse health outcomes in adolescents in rural Haiti.

**Supplementary Information:**

The online version contains supplementary material available at 10.1186/s12905-024-03136-6.

## Introduction

Young women in Haiti experience high pregnancy rates, with 53 annual births per 1,000 adolescents (ages 15–19) [1] in 2021, and 10% reported having children or being currently pregnant in 2017, many of which are unintended [2–4]. Unmet need for contraception contributed to an estimated 413,000 unintended pregnancies in Haiti in 2019 [5], which are in turn associated with adverse outcomes such as increased rates of unsafe abortions and increased risk of maternal complications or mortality [6]. Haiti’s maternal mortality rates remain disproportionally high with 480 deaths per 100,000 live births, compared to neighboring Dominican Republic and Cuba (95 and 36 and per 100,000 live births respectively) [7]. A study of 172 countries found at least 30% of maternal deaths, 45% specifically in Haiti, could be prevented by meeting needs for unmet contraception use [8]. Closing the gap between contraception needs and access could result in numerous positive outcomes for adolescents in Haiti, who report unique physical, economic, and psychosocial challenges with unintended pregnancies and subsequent transition to parenthood [9, 10].

Healthcare providers (HCPs) play an important role in facilitating contraception access and use. Though World Health Organization guidelines recommend pregnancy prevention care including autonomy supportive contraceptive when desired [11], many HCPs do not provide this evidence-based care for adolescents [12]. HCPs may have moral or social reservations about discussing contraception with adolescents [13, 14] and also may not have the knowledge or training to counsel about the full range of contraception options, particularly long acting reversible contraception (LARC). To our knowledge only one study has examined HCP contraception perceptions in Haiti. The study focused on postpartum women living in Northern Haiti and identified provider-level barriers, including insufficient knowledge and lack of time or privacy and highlighted younger women as particularly vulnerable [15]. No studies have examined the perceptions of HCPs regarding contraception use among adolescent women in Haiti, particularly in rural communities. As Haitian policy requires public health clinics to provide free contraception services [2], it is critical to examine what barriers impact HCP provision of this critical care.

The goal of this mixed-methods study was to describe HCPs’ attitudes, subjective norms and perceived behavioral control related to contraceptive care for adolescents (ages 14–18) in rural Haiti.

## Methods

### Study design

**Fig. 1:**
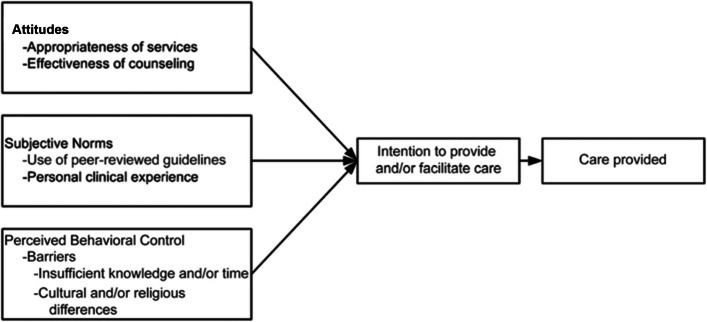
The TPB is adapted to model the provision of sexual and reproductive healthcare to adolescents and young adults

We conducted a cross-sectional survey and qualitative interviews of HCPs in rural Haiti from August 2021 to March 2022. Surveys and interviews were based on the Theory of Planned Behavior (TPB), a theoretical framework which asserts that behavioral intention is driven by attitudes (e.g., beliefs and values), subjective norms (e.g., influence of important peers, personal experience), and perceived behavior control (e.g., barriers and facilitators of the behavior) [16–18]. The TPB is a comprehensive model that has been widely used to describe healthcare provider influence on health behaviors that are driven strongly by behavioral intention, including sexual health behaviors and contraception use (Fig. 1).

Inclusion criteria for HCPs included clinical staff (e.g., nurses, nurse midwives, physicians, other) who engage in reproductive health care at a clinic or hospital affiliated with one of our partner organizations, Global Birthing Home Foundation and Hospital Albert Schweitzer. Global Birthing Home Foundation is a free-standing maternal health center in the Southern Department of Haiti staffed by Haitian nurse midwives who offer contraceptive care, as well as prenatal, labor and delivery and postnatal care. High risk patients and pregnancies are referred for obstetric/gynecologist care at the regional hospital. Hospital Albert Schweitzer is a community hospital in the Central Department of Haiti that offers reproductive and obstetric care by obstetric/gynecologist physicians and nurse midwives. This hospital operates several community health centers in the region that also offer contraceptive care. Global Birthing Home Foundation and Hospital Albert Schweitzer have no affiliation with one another. This study was conducted concurrently with our previously published study on Haitian adolescents and young adults (AYAs, ages 14–24) perspectives on contraception [10].

The study was reviewed (including study protocol, all study documents and verbal consent process) and approved by the institutional review board at the affiliated academic institution, Children’s Mercy Hospital, Kansas City, MO, in the United States and by an administrative leader at each study site (Global Birthing Home Foundation, Hospital Albert Schweitzer) according to local procedures in Haiti. Study materials were first reviewed by our study team, including our co-investigator and bilingual university-level translator (DC) who is a Haitian young adult female and native to the community where Global Birth Home Foundation is located. Her feedback informed changes to the survey and interview guides to ensure the cultural appropriateness of our study materials. All study materials were created in English and translated into Haitian Creole by our previously described co-investigator (DC). Translated documents were reviewed and revised by the local research team in Haiti and with three HCPs representative of the sample to ensure accuracy and comprehension. Verbal consent and data collection were conducted in Haitian Creole.

### Procedures

The research team included two local research assistants (i.e., young adult females fluent in Haitian Creole) who were trained in study procedures (i.e., verbal consent, survey and interview administration, data collection). The research assistants worked with staff at each healthcare facility to recruit a convenience sample of eligible HCPs. They approached eligible staff at their place of work at a convenient time during the workday and obtained verbal consent to participate. Due to procedural error, we did not capture the number of declinations and reasons for declination. Participants were given the choice to privately self-administer or to complete the survey administered by the research assistants privately on a tablet with no identifiable information collected. Study data were entered and managed using Research Electronic Data Capture (REDCap) hosted at Children’s Mercy Hospital [19, 20]. Upon survey completion, a sub-sample of HCPs were then asked to complete a semi-structured audio-recorded interview until reaching a sample size expected to achieve thematic saturation [21]. The survey and interview took approximately thirty minutes each to complete. All HCP participants were given a phone card worth $5 US upon completion of study activities (i.e., survey, or survey and interview).

### Survey instrument

We developed a survey using previously validated instruments (more details below) to assess HCP demographics, clinical experience and practice behaviors, as well as 46 items assessing TPB [5] constructs including attitudes, subjective norms, and perceived behavioral control regarding contraceptive care for adolescents (see appendix) [22–24].

#### Demographics and clinical experience

We used multiple choice questions to assess demographic information including age and training background. To assess practice behaviors, participants reported their estimated frequency of obtaining a sexual history when caring for adolescents using a 5-point Likert scale (“always,” “very often,” “sometimes,” “rarely,” or “never”) and their estimated frequency of caring for adolescents with a comorbid condition associated with possible pregnancy complications (categorized as 0, 1–5, 6–10, 11–20, and > 20%). We also assessed interest in further education in taking a sexual history and contraception provision using a 5-point Likert scale (“extremely,” “moderately,” “somewhat,” “slightly,” or “not at all”).

#### Attitudes

To assess attitudes, participants reported how strongly they agreed or disagreed with statements about the provision of sexual health services that are appropriate to offer adolescents in their clinical settings (e.g., counseling for pregnancy prevention, safer sexual behaviors, or condom use).

#### Subjective norms

To assess subjective norms, one survey item asked about sources of information that inform HCP’s reproductive care for adolescents (“Which of the following do you consider when providing reproductive care to an adolescent in your practice?” (e.g., informal guidelines, national guidelines).

#### Perceived behavioral control

To assess perceived behavioral control we focused on multi-level barriers. To assess this, participants responded to survey items about barriers on the levels of individual provider (e.g., insufficient knowledge), patient (e.g., fear of disclosure to parent), interpersonal (e.g., clinical-patient gender differences) and system (e.g., lack of staff experience) using a 5-point Likert scale (“strongly agree,” “agree,” “neutral,” “disagree,” or “strongly disagree”).

### Semi-structured interview

Interviews were used to supplement our quantitative findings, specifically to elicit deeper individual HCP perspectives on contraception counseling for adolescents. The guides (see appendix) included open-ended questions based on the TPB constructs.

For example, questions exploring *attitudes* included: “In general, how do providers at this clinic feel about talking about providing contraception to adolescent women?", “What do you think impacts adolescents’ decisions to use birth control?” and “In your opinion, what should be the role of healthcare providers be in reducing unintended pregnancy among adolescents and young adults?”. Questions on *subjective norms* included: “Who are the people or groups of people that influence your practice of care?”, “What is the role of your colleagues, supervisors, donors, and of the ministry of health or national guidelines?’’. *Perceived behavioral control* was explored with questions including: “In your opinion, what are the biggest challenges in discussing sexual health and pregnancy prevention with adolescent female patients?” and “In your opinion, what needs to change so that this clinic can better address the sexual health needs of adolescents?”.

### Data analysis

For quantitative analysis, survey findings were translated from Haitian Creole back to English and descriptive statistics were used to present the categorical data as proportions. There were no missing data. All statistical calculations were conducted using SPSS, Version 20 or SAS software v 9.4 (SAS Institute, Cary, NC, USA).

For qualitative analysis, interview audio-recordings were transcribed and translated from Haitian Creole to English. We used an iterative process to identify emergent themes within and across interviews. Interview transcripts were uploaded into Dedoose software (Version 4.12) for analysis. A coding tree was developed based on the interview guides and periodically revised to include relevant inductive codes as emergent themes arose. The first three interviews were coded together by three members of the study team (Masonbrink, Hurley, Schuetz) to develop mutually agreed upon definitions for each code and to establish examples of each code. Codes were reviewed and revised, and the interviews were again coded by the same team members. Any disagreements in coding were resolved by consensus. Each interview was then coded separately by 1–2 members of the study team. Upon reaching thematic saturation, the three team members met to discuss the results; again, any disagreements in coding were resolved by consensus. Memos of coding decisions were kept to provide consistency in coding as the coding progressed. The coding team summarized coding outputs, synthesizing major themes according to TPB constructs.

## Results

### Quantitative findings

#### Demographics and clinical experience

Among our 58 respondents, a majority were female (*n* = 52, 90%), were either 30 to 39 (*n* = 19, 33%) or 40 to 49 (*n* = 25, 43%) years old and approximately half were nurses (*n* = 31, 53%) and 17% (*n* = 10) were medical doctors (Table 1). Most HCPs reported always (*n* = 16, 28%) or very often (*n* = 21, 36%) obtaining a sexual history when caring for adolescent patients. Most HCPs (*n* = 51, 88%) estimated <  = 10% of their adolescent patients have a health condition (e.g., diabetes mellitus, depression) that could cause pregnancy complications.

**Table 1:** Healthcare provider characteristics

Characteristic	**HCP Characteristics (** ***N*** ** = 58)**	
	Categories	n (%)
Sex	Male	6 (10)
	Female	52 (90)
Age category (years)	20–29	7 (12)
	30–39	19 (33)
	40–49	25 (43)
	50–59	6 (10)
	60 +	1 (2)
Years since completion of most advanced clinical training	< 5	16 (28)
	5–10	19 (33)
	11–20	15 (26)
	> 20	7 (12)
	Did not answer	1 (2)
Level of Training	Nursing	31 (53)
	Nurse midwife	6 (10)
	Medical doctor	10 (17)
	Other	11 (19)

#### Attitudes

**Fig. 2:**
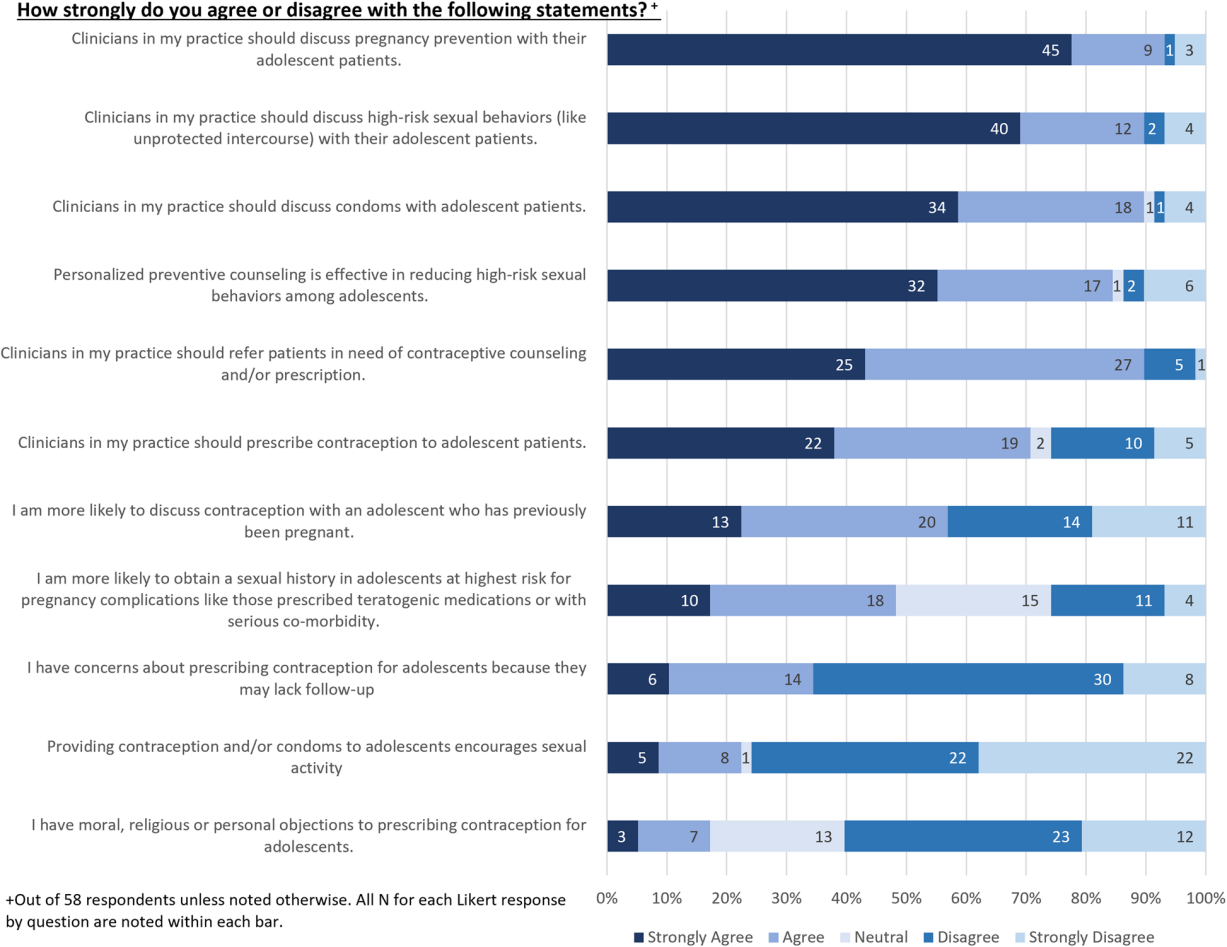
HCP Attitudes about Contraception Provision for Adolescents

HCP attitudes about contraception provision for adolescents are described in Fig. 2. A majority of HCPs agreed or strongly agreed that clinicians should discuss pregnancy prevention (*n* = 54, 94%) and high-risk sexual behaviors (*n* = 52, 90%) with their adolescent patients. Similarly, most agreed or strongly agreed (*n* = 52, 90%) that clinicians should discuss condoms with their adolescent patients. A majority agreed or strongly agreed (*n* = 49, 84%) that personalized preventive counseling is effective in reducing high-risk sexual behaviors among adolescents.

#### Subjective norms

Most HCPs reported they consider informal (e.g., institution specific) guidelines (*n* = 25, 43%) or published guidelines from national medical organizations (*n* = 22, 38%) when providing reproductive care for adolescents.

#### Perceived behavioral control (barriers)

**Fig. 3:**
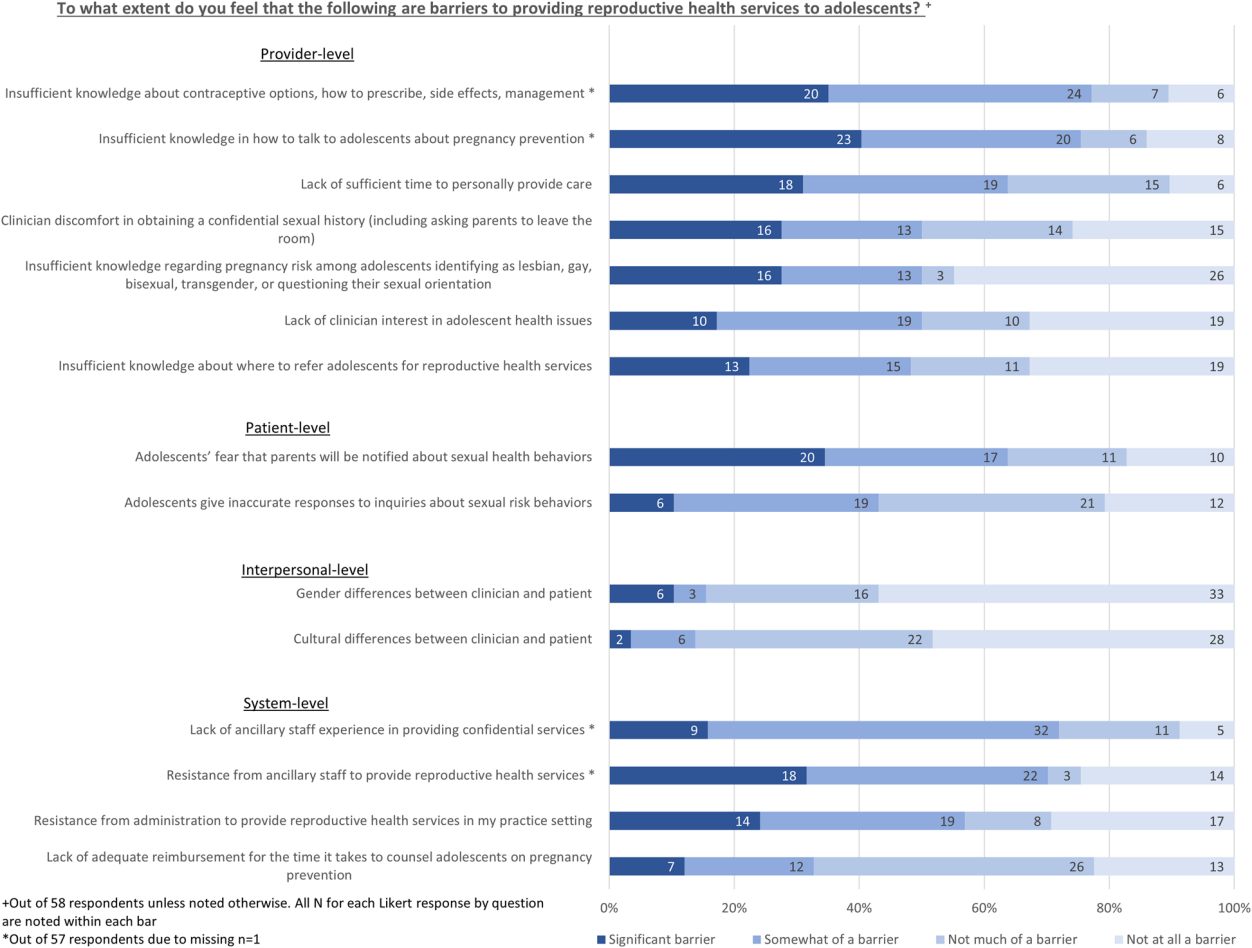
HCP Perceived Barriers to Contraception Provision

HCP perceived barriers to contraception provision for adolescents are described in Fig. 3. The most frequently cited provider-level barriers (i.e., significant or somewhat of a barrier) to contraceptive care for adolescents included insufficient knowledge about contraceptive management (*n* = 44, 77%), insufficient knowledge in how to talk to adolescents about pregnancy prevention (*n* = 43, 75%), and lack of sufficient time to personally provide care (*n* = 37, 64%). Patient-level barriers (i.e., rated as significant or somewhat of a barrier) included concerns that adolescents fear parents will be notified about sexual health behaviors (*n* = 37, 64%) and that adolescents give inaccurate information about their sexual behaviors (*n* = 25, 43%). System-level barriers included resistance to provide reproductive health services in the practice setting from ancillary staff (*n* = 40, 71%) and administration (*n* = 33, 57%).

To decrease barriers, a majority reported they were “very interested” in further training regarding contraception management (*n* = 51, 88%), adolescent consent and confidentiality (*n* = 42, 72%) and taking a sexual history (*n* = 40, 69%). Given this further training, 49 (86%) reported they would be likely or extremely likely to increase provision of these services.

#### Qualitative interview findings

Seventeen HCPs participated in qualitative interviews with a mean age of 39.6 (standard deviation [SD]: 9.9) years and 11.3 (SD: 5.7) mean years of experience. Of these 10 were nurses, 3 were nurses aids, 2 were midwives, and 2 were lab technicians. Our qualitative findings commonly supported our quantitative findings, while also revealing additional factors. Table 2 summarizes themes and provides illustrative quotes related to each TPB construct (i.e., attitudes, subjective norms, perceived behavioral control) regarding barriers and facilitators to contraceptive care delivery for adolescents.

**Table 2:** Qualitative interview illustrative quotes

TPB Construct and Interview Themes	Illustrative Quote
***Beliefs*** *HCPs are concerned about the rate of unintended pregnancies in AYAs*	A lot of adolescents are dealing with unintended pregnancy, and they can’t take care of themselves and their babies. My concerns are the fact we don’t have enough funds to help the young adolescent that are in great need for care, we can’t do much to educate them about sexual health. (nurse, age 44 years)
	I am very concern(ed) because a lot of adolescents have been practicing early sex which led to early pregnancies which is a disruption in their education. (nurse aid, age unknown)
*Importance of offering contraception education as a primary HCP role*	Their roles should be educating them about sexual health and birth control, the negatives impact of early pregnancies in teens…We don’t prescribe birth control, we have educational class(es) about it, but they are one that choose in the end whether to have it or not. (nurse, age unknown)
	Their roles should be mobilization, education, from door to door, in all the schools so that adolescents know what birth control is. (nurse, age 44 years)
*HCPs believe educating AYAs will reduce unintended pregnancies*	The ones that are educated about it, they do it, and they like the fact they have control over their body and unintended pregnancies. (nurse, age unknown)
	The biggest health issues are the fact a lot of adolescents nowadays are dealing with unintended pregnancy, and I think it’s because of lack of sexual education. (nurse, age unknown)
*HCPs are supportive of LARC methods*	I think they are great method because they last for a long time, implant (5–7 years,) and IUD (10-12 years.) (nurse, age 28 years)
	They are great methods because they last longer, and people won’t have to worries about getting pregnant…Their benefits are socio-economic, the fact you don’t have to worry about unintended pregnant for a long period of time and you can plan better your future, it also help with taking break between the pregnancies. (nurse, age unknown)
	I haven’t administered the IUD yet, but I am very comfortable with the subdermal implant and my patients are very happy using it. (midwife, age 32 years)
***Perceived Behavioral Control (Barriers)*** *Lack of AYA education about sexual health and contraception*	I think it is because they are not educating about sexual health. my 12–13 years old patients don’t know anything about preventing pregnancy, they have sex just because they heard about it and wanted to try it and ending up pregnant. (midwife, age unknown)
	The barriers are educational ones, because they are not educated about birth control, they fear their side’s effects, they are also worried of what people might think of them if they use birth control. (nurse, age 29 years)
*AYA misconceptions about birth control use and side effects*	There is that false belief in their heads about birth control, they believe using birth control before having kids will prevent them from having kids in the future (making them sterile). (nurse, age 44 years)
	They think that birth control would make them loose or gain weight, and making their period last longer. (lab technician/ health promoter, age 35 years)
	I think it’s the rumors they have been hearing about birth control. They think they can’t use birth control before having kids, it will destroy their white blood cells, they even think using condom will give diseases because it greasy. (nurse aid, age unknown)
*Lack of parental knowledge and support*	The biggest challenge is for their parents to understand that talking to their adolescent about sexual health should be totally normal. (lab technician, age unknown)
	Their parents aren’t educated about sexual health; therefore, this subject is prohibited to talk about to their kids. (nurse aid, age 46 years)
	One of the biggest issues the young in this community are facing the lack of sexual education. It is a taboo subject for parents to talk about with their kids. (nurse, age unknown)
*Importance of peer opinion and experiences*	They think there is more disadvantages than advantages, if one is telling about her side effects about a type that she uses her friends will be more likely to think that’s a red flag, therefore they will not want to use it. (midwife, age unknown)
	Some of their peers that have had some normal side effects while using birth control discourage the ones that aren’t using birth control yet. (nurse, age 55 years)
***Future Directions and Needs*** *Need for more contraception supplies and training*	I think we need more equipment to administer the subdermal methods, we also need to train more staff about how to administer them. (nurse, age 28 years)
	There are some birth control methods that we don’t have in our clinic, such as IUD, etc. I would like for us to have whatever types the patients want. (nurse, age 29 years)
	They need to train more people to go and work in their communities, because most of the villages don’t have access to healthcare, therefore they don’t know anything about what birth control is, and how to prevent unintended pregnancy. (nurse, age 44 years)
*Importance of providing contraceptive care and education in a private, youth-friendly and accessible setting*	I think it will be useful to have more places for people to go for birth control, mostly in deep villages, because they are less likely to access to this kind of care. (nurse, age 28 years)
	They are worried about that the nurse or other people who recognize them in the PF section might go and tell their business in their neighborhood, for that reason they prefer not to come for birth control in clinics or hospital. (nurse aid, age 46 years)
	They need to be educated about birth control in schools and churches, they also need to organize a specific club for to teach them about the negatives impacts of early pregnancies. (nurse, age unknown)
	I think MSPP (i.e., the Haitian Ministry of Health) should be implementing the PF [family planning] program in school from 9th grade as a class so that they know what sexual health and birth control are. (nurse, age 29 years)

### Attitudes

In interviews, many HCPs named pregnancy as one of the biggest health issues facing adolescents in their community. For example, one nurse stated “I am concerned about the frequency in which young adolescent women are getting pregnant nowadays. They can’t provide for these babies since they can barely provide for themselves, and oftentimes those babies are often suffering from malnutrition” (nurse, age 32 years). A majority of HCPs reported they feel they play a vital role in providing adolescents contraception education. Many stated that they believe educating youth, and their parents, on contraception is important to prevent unintended pregnancy; for example, a nurse stated “Their (HCP’s) roles should be to educate adolescent women about sexual health and birth control, they also need to educate their parents about it” (nurse, age 29 years). A minority of HCPs felt that promoting abstinence was important in pregnancy prevention. HCPs were also supportive of LARC for their adolescent patients, for example one participant stated “I think these methods are the best because they last a long time, that means people won’t have to worry about that them for a long time…I haven’t administered the IUD yet, but I am very comfortable with the subdermal implant and my patients are very happy using it” (midwife, age 32 years).

### Subjective norms

When asked “Who are the people or groups of people that influence your practice of care?”, HCPs most commonly stated their administrative or clinical leaders (i.e., supervisor, director, nurse in charge) influenced their practice of care. These participants further stated they felt their administration’s role was to keep everything running smoothly and ensure staff had adequate training.

### Perceived behavioral control (barriers)

Similar to the survey findings, during interviews many HCPs noted concerns about maintaining privacy and confidentiality while providing contraceptive care for adolescents. HCPs acknowledged that discussions about pregnancy and contraception with adolescents are sensitive topics in part due to community judgment; for example one participant stated “It is a sensitive conversation because everyone has their own opinions about pregnancy, they tend to be judgmental about pregnancy among young adolescents” (nurse, age 44 years). Additionally, HCPs reported that adolescents lack sufficient education about birth control. One participant stated “I am concerned because clearly no work has been done really to educate adolescents about sexual health. If they were educated about sexual health, I am sure they would put their knowledge to use and have safer sex” (nurse, age unknown). HCPs also noted further concerns about adolescents’ misconceptions about contraception. For example, one nurse stated, “They think they are too young for using birth control, they also think it will destroy all their white blood cells if they aren’t sexually active” (nurse, age unknown). Interestingly some HCPs themselves reported misconceptions about certain contraception methods; for example, one stated “Personally I think they string [IUD] might attract bacteria and causes some infections” (nurse, age unknown).

An additional barrier included concerns about lack of parental support. For example, one participant stated “The biggest challenge is their parents, they don’t want you as a healthcare provider to talk to their kids about birth control, they think you are encouraging them to go and have sex” (nurse, age 28 years). HCPs also noted that peer opinion and experiences influence adolescents’ perceptions about contraception use; for example one participant stated “Their peers impact them a lot, if one within a group of friends is using birth control and encounter some issues, that one will tell the group how birth control in general is troublesome and the rest of them will more likely stay away from it without even trying it” (nurse aid, age 48).

Further, most HCPs stated that they are comfortable providing contraception counseling to adolescents and remarked they are knowledgeable to provide education on the types and side effects. This finding differed from our survey results as most HCPs noted concerns insufficient knowledge about contraceptive options, how to prescribe, and side effects as significant barriers.

To decrease barriers, many HCPs expressed the desire for further training regarding contraceptive care for adolescents, some also noted the need for training for health promoters or community health workers. Some noted the need for more contraception options and consistent supplies, saying “I think they need to have all the different types of birth control here so that young women have more choices, have them always available not running on shortage like we always do here” (nurse, age unknown). Additionally, HCPs noted the importance of providing contraceptive care in youth-friendly settings. For example, one HCP stated “I think they need to create a space specially to educate the adolescents about sexual health, because putting them in a space with adult patients that are probably the same age as their moms to discuss sexual health makes them uncomfortable” (nurse, age unknown).

## Discussion

In this multi-site, mixed-methods study, we used the TPB framework to describe HCP perspectives on contraceptive care for adolescents in rural Haiti. A majority of HCPs shared concerns about unintended pregnancy and agreed they play an important role in pregnancy prevention and contraception provision for adolescents in their community. Despite international guidelines to provide sexual and reproductive health services for youth [11], more than one third of participants reported only sometimes or rarely obtaining a sexual history from their adolescent patients. While HCPs were supportive of contraceptive care for adolescents, we identified numerous actionable barriers to provision of this care.

Similar to past literature, in our study most HCPs expressed concerns about insufficient knowledge and time to provide this care [14, 25]. Interestingly, during interviews many HCPs reported they felt comfortable and knowledgeable to provide contraception for adolescents. This discrepancy may be in part related to social desirability bias that influenced answers during in-person interviews rather than anonymous self-reporting during the survey. However, in both surveys and interviews, a large majority of HCPs desired further training on contraceptive care for youth. HCPs noted concerns about liability, and parental, community and peer judgement as barriers to contraception provision, which has been previously described [26]. Importantly, this finding also aligns with results from a previously published study as many youth also endorsed concern about judgement by peers, parents and HCPs as a significant barrier to contraception [10]. Thus, community outreach and interventions to improve parental and community contraception knowledge and support could improve contraceptive care for adolescents [27, 28]. HCPs also reported concerns about privacy and adolescent patients giving inaccurate information about their sexual behaviors. Given these concerns, many HCPs reported interest in receiving further training on consent and confidentiality and many also endorsed the importance of contraception provision in youth-friendly settings. We recently published AYA perspectives on contraception in Haiti, those results similarly highlighted the need for a youth-friendly approach to contraceptive care [10].

HCPs reported their contraception clinical practice is influenced by national or informal guidelines as well as by their clinical leadership (i.e., supervisors, directors). However, HCPs also noted concerns about resistance from ancillary staff and administration to contraceptive care. Studies in similar settings have noted concerns about the impact of national policies limiting provision of contraception care for youth, particularly those who are unmarried [14]. Additionally, lack of support and resources from clinical administrative staff, is also a known barrier to contraceptive care, including certain methods (e.g., LARC) [29–31]. Further exploration is needed to better understand the influence of national policies as well as the perspectives and impact of clinical leadership and administration on contraception care for youth.

International guidelines support provision of patient-centered contraceptive care for youth, including LARC [11]. We found HCPs were supportive of offering LARC (ie., subdermal implant, intrauterine device [IUD]) for adolescents, however, many HCPs noted a lack of LARC training and supplies. Additionally, some HCPs reported misconceptions about IUD side effects. This finding aligns with our previously published study, as many youth also similarly reported misconceptions on contraception side effects, including IUDs [10]. Thus, future efforts to provide LARC training, resources, and supplies are needed to increase access to these highly effective contraception methods.

Our findings should be viewed in light of the following limitations. Our enrollment consisted of a convenience sample of HCPs and therefore may be at risk for sampling bias. Reasons for declination were not collected so we were unable to compare between those who participated and declined. We enrolled a relatively small sample size (*n* = 58) though we enrolled across two sites to increase generalizability. While our findings may not be generalizable to other HCPs or clinical settings in Haiti, the demographics (e.g., age, sex, years of experience) of our study participants are similar to other HCP studies in Haiti [32, 33]. Lastly, due to the sensitivity of the topic there is a risk of inaccurate reporting as well as social desirability bias especially during interviews. Our team attempted to mitigate this risk by working with trusted community members and ensuring private tablet-based data collection.

While HCPs support contraceptive care we revealed a number of actionable barriers to improve care for adolescents in rural Haiti. Efforts to increase HCP knowledge and training on contraceptive care for adolescents, as well as resources to offer all contraception methods, including LARC, are needed. Further, HCPs highlighted the importance of providing contraception counseling in private, youth-friendly accessible settings, as well as community and parental engagement to increase education and support for contraception for adolescents in Haiti. Given the high rates of unintended pregnancy among adolescents in Haiti largely due to high unmet contraception needs, efforts to improve contraception access in this population are critically needed.

### Supplementary Information

Supplementary Material 1.

Supplementary Material 2.

## Data Availability

The datasets generated and/or analyzed during the current study are not publicly available due to the sensitive topic and lack of consent granted from participants to share the data but are available from the corresponding author on reasonable request. Additionally, the study protocol, analysis plan, informed consent forms and clinical study report will be made available upon request by any researchers who provide methodologically sound proposal beginning 3 months and ending 5 years following article publication.

## Abbreviations

HCP: Healthcare provider
LARC: Long acting reversible contraception
TPB: Theory of planned behavior
IUD: Intrauterine device
