# Supplementary material for: Barriers to pregnancy prevention for adolescents in rural Haiti: perceptions of healthcare providers
Source: BMC Womens Health. 2024 May 22;24:305. doi: 10.1186/s12905-024-03136-6 (PMC11110254; doi:10.1186/s12905-024-03136-6)
Supplement: Supplementary file 1 — Supplementary Material 1. [file 12905_2024_3136_MOESM1_ESM.docx]

**Interview Guide:**

**Healthcare providers**

*[After informed consent]:* Thank you for agreeing to talk with us today. We are trying to develop resources for young women who live in this area to support them making informed decisions about pregnancy and pregnancy prevention. Your thoughts and experiences about these issues will really help us.

I just want to remind you that there are no right or wrong answers to the questions, we are really just interested in your own personal opinions. And remember, if there is ever a question you don’t feel comfortable answering, that’s perfectly fine. If it’s on the iPad just click “declined to answer”. If it’s something I ask you in the interview, just let me know and we can skip it.

*[After participant finishes iPad questions]:* Thanks so much for answering these. Do you mind if we start the recording and get into the interview?

*[Notes to interviewer: Bullet points are optional probes. Select as appropriate. Utilize general probes such as “tell me more” or “what do you mean by that” as needed to expand the discussion.]*

These first questions are about your role here at the clinic.

1. I understand you are a [current position] and you’ve been with [organization], Can you briefly describe your role here?
   - What other roles have you filled at [organization]?
   - What sort of work did you do prior to joining [organization]? / What other types of facilities have you practiced in?
   - Probes: years of experience
   - How old are you?
2. How often do you provide care for adolescents?

- What type of care to you provide to adolescents?

Now I’d like to hear your thoughts about adolescent health in this community

1. What do you feel are the biggest health issues facing adolescents in your community?

When we say adolescent, we mean youth aged 14-18 years who function independently and engage in typical activities and behaviors for their age.

1. To what extent are you concerned about unplanned pregnancy among adolescents in this community?
   - What are your concerns?
   - How do your adolescent patients typically feel about pregnancy?
2. What barriers do adolescent women face in preventing pregnancy?
   - How do these compare to barriers faced by older women in the community?
   - What is it like for adolescents to get sexual health care in this community?
   - What are ways to make it easier adolescents to prevent pregnancy?
3. What do you think impacts adolescents’ decisions to use birth control?
   - How, if at all, is this different for younger adolescents, for instance those age 14 to 16 years old?
   - How do adolescent women feel about contraception?
   - What are the challenges they face in using contraception effectively?
   - How do the feelings of their partner factor into their contraception use?
   - How do the feelings of their peers factor into their contraception use?
   - How do the feelings of their partners factor into their contraception use?
   - How common is intimate partner violence among adolescents in this community? How does it affect the use of birth control?
   - How common is sex trafficking or exchanging sex for drugs or other things in this community? How does it affect the use of birth control?
4. In your opinion, what is the best way for a young women to prevent pregnancy?
   - For young women who want to use contraception, what do you think is th best method?

- What types of contraception do your young patients typically prefer?

Now I’d like to learn more about your thoughts and experiences providing reproductive health care to adolescent women.

1. In your opinion, what should be the role of health care providers be in reducing unintended pregnancy among adolescents and young adults?
   - How do your ideas about reducing unintended pregnancy among adolescents compare to those of your colleagues?
   - What conversations have you had with your colleagues about adolescent pregnancy?
   - How does the leadership at your clinic feel about addressing adolescent pregnancy?
   - What about other local providers outside of your clinic, how concerned are they about this issue?
2. What are your thoughts about discussing sexual health and pregnancy prevention with adolescent female patients?
   - How comfortable are you discussing these issues with your adolescent patients?
   - How much do you typically know about your young patients’ sexual behaviors?
   - How often do young women initiate a conversation about their reproductive health needs?
   - How often do you initiate the conversation? How do you go about it?
   - How often do you take a sexual history for your adolescent patients? How comfortable are you doing so?
   - How often do you discuss relationship problems, including intimate partner violence (IPV), with your adolescent female patients? How comfortable are you doing so?
   - What is your experience providing care for young women who have been involved in sex trafficking or exchanging sex for drugs or other things?
3. In your opinion, what are the biggest challenges in discussing sexual health and pregnancy prevention with adolescent female patients?

What challenges have you encountered in discussion sexual health with adolescents?

- - What strategies do you use to overcome these challenges?
  - What ideas do you have about how to make these conversations easier?

Let’s focus the conversation a little more on contraception.

1. What is your experience with providing contraception to women of any age here at your clinic?
   - What types of contraception are offered here at the clinic?
   - Walk me through how you guide a woman in choosing contraception.
   - What types of contraception are you most comfortable offering? Which are you least comfortable offering?
2. In general, how do providers at this clinic feel about talking about providing contraception to adolescent women?
   - What are your personal feelings?
   - What do your colleagues think about prescribing contraception for adolescents? What about your supervisor?
     1. What would make them more comfortable?
   - How comfortable are your administrators with providing these services at your institution/organization?
     1. What would make them more comfortable?
   - What other limitations or barriers for prescribing contraception for adolescents exist at your workplace?
3. What is your own experience with providing contraception to adolescent women here at your clinic?
   - Walk me through how you guide an adolescent woman in woman in choosing contraception.
   - What do you consider when talking about contraception to an adolescent patient who has already had a baby or is post-partum?
4. What are your thoughts about long acting reversible contraception (such as IUD or subdermal implants)?
   - What do you know about these methods?
   - What do you see as the benefits of long acting contraception, like the IUD? What about the implant?
   - What do you see as the harms of using an IUD? What about the implant?
   - What do you think about offering these types of long acting contraception methods to adolescents and young adults in your clinic?
5. Tell me about more about your experience with LARCs
   - What types of long-acting reversible contraception or LARC (IUDs and subdermal implants) available in your clinical setting?
   - What training have you received on these contraceptive methods?
   - How comfortable are you in administering LARC?
     1. How comfortable would you be learning to administer LARC?
   - How comfortable are your colleagues in administering LARC?
     1. Who would be the best person in your clinical setting to be trained to administer LARC?

Lastly, I’d like to learn a little bit about how decisions are made for what health care services should be offered at this clinic.

1. Who are the people or groups of people that influence your practice of care?

- What is the role of your colleagues?
- What is the role of your supervisors?
- What is the role of donors?
- What is the role of the ministry of health or national guidelines?

1. In your opinion, what needs to change so that this clinic can better address the sexual health needs of adolescents?
   - What would need to happen to put these changes in place?
   - Who would need to make these decisions?
   - In your opinion, would it ever be possible to providing contraception counseling and medications as part of routine care for adolescents? What needs to happen in order to put this in place?
2. Thank you so much for sharing your thoughts with us. Is there anything else that we should have asked you about or anything else you’d like to tell us?
3. Do you have any questions for me before we go?
