# Supplementary material for: Barriers to pregnancy prevention for adolescents in rural Haiti: perceptions of healthcare providers
Source: BMC Womens Health. 2024 May 22;24:305. doi: 10.1186/s12905-024-03136-6 (PMC11110254; doi:10.1186/s12905-024-03136-6)
Supplement: Supplementary file 2 — Supplementary Material 2. [file 12905_2024_3136_MOESM2_ESM.docx]

**Healthcare Provider Perspectives Survey Instrument**

Sex:

Male

Female

Prefer not to answer

What is your age?

20-29 years

30-39 years

40-49 years

50-59 years

60+ years

Years since completion of most advanced clinical training:

<5

5-10

11-20

>20

What level of training have you completed? (may select more than one)

Nursing

Nurse midwife

Medical school

Other

When caring for an adolescent (14-18 years) for the first time how often would you typically obtain a sexual history?

Always

Very often

Sometimes

Rarely

Never

For the rest of this survey, when we refer to adolescents, we mean youth aged 14-18 years who function independently and engage in typical activities and behaviors for their age.

How strongly do you agree or disagree with the following statements?

Circle ONE response for EACH item.

|  | Strongly Disagree | Disagree | Neutral | Agree | Strongly Agree |
| --- | --- | --- | --- | --- | --- |
| Clinicians in my practice should discuss high-risk sexual behaviors (like unprotected intercourse) with their adolescent patients. | 1 | 2 | 3 | 4 | 5 |
| Clinicians in my practice should discuss pregnancy prevention with their adolescent patients. | 1 | 2 | 3 | 4 | 5 |
| Clinicians in my practice should prescribe contraception to adolescent patients. | 1 | 2 | 3 | 4 | 5 |
| Clinicians in my practice should discuss condoms with adolescent patients. | 1 | 2 | 3 | 4 | 5 |
| Clinicians in my practice should refer patients in need of contraceptive counseling and/or prescription. | 1 | 2 | 3 | 4 | 5 |
| It is appropriate to dispense an emergency contraceptive pill in my clinical practice setting. (need to determine if acceptable to ask about EC with our Haitian collaborators) | 1 | 2 | 3 | 4 | 5 |
| Providing contraception and/or condoms to adolescents encourages sexual activity. | 1 | 2 | 3 | 4 | 5 |
| I have moral, religious or personal objections to prescribing contraception for adolescents. | 1 | 2 | 3 | 4 | 5 |
| I have moral, religious or personal objections to prescribing an emergency contraception pill. (need to determine if acceptable to ask about EC with our Haitian collaborators) | 1 | 2 | 3 | 4 | 5 |
| I have concerns about prescribing contraception for adolescents because they may lack follow-up | 1 | 2 | 3 | 4 | 5 |
| I have concerns about prescribing emergency contraception for adolescents because they may lack follow-up (need to determine if acceptable to ask about EC with our Haitian collaborators) | 1 | 2 | 3 | 4 | 5 |
| Personalized preventive counseling is effective in reducing high-risk sexual behaviors among adolescents. | 1 | 2 | 3 | 4 | 5 |
| I am more likely to discuss contraception with an adolescent who has previously been pregnant. | 1 | 2 | 3 | 4 | 5 |
| I am more likely to obtain a sexual history in adolescents at highest risk for pregnancy complications like those prescribed teratogenic medications or with serious co-morbidity. | 1 | 2 | 3 | 4 | 5 |
| I am concerned about liability with respect to prescribing contraception to adolescents in my practice. | 1 | 2 | 3 | 4 | 5 |
| I am concerned about side effects of hormonal contraception in adolescents. | 1 | 2 | 3 | 4 | 5 |
| I am concerned that parents of my adolescent patients will be upset if I ask to speak privately with their child. | 1 | 2 | 3 | 4 | 5 |
| I am concerned that parents of my adolescent patients will be upset if I discuss pregnancy prevention with their child. | 1 | 2 | 3 | 4 | 5 |

To what extent do you feel that the following are barriers to providing reproductive health services to adolescents?

Circle ONE response for EACH item

|  | Significant barrier | Somewhat of a barrier | Not much of a barrier | Not at all a barrier |
| --- | --- | --- | --- | --- |
| Cultural differences between clinician and patient | 1 | 2 | 4 | 5 |
| Gender differences between clinician and patient | 1 | 2 | 4 | 5 |
| Lack of clinician interest in adolescent health issues | 1 | 2 | 4 | 5 |
| Clinician discomfort in obtaining a confidential sexual history (including asking parents to leave the room) | 1 | 2 | 4 | 5 |
| Adolescents give inaccurate responses to inquiries about sexual risk behaviors | 1 | 2 | 4 | 5 |
| Lack of adequate reimbursement for the time it takes to counsel adolescents on pregnancy prevention | 1 | 2 | 4 | 5 |
| Insufficient knowledge in how to talk to adolescents about sexual risk reduction | 1 | 2 | 4 | 5 |
| Insufficient knowledge in how to talk to adolescents about pregnancy prevention | 1 | 2 | 4 | 5 |
| Insufficient knowledge about contraceptive options, how to prescribe, side effects, management | 1 | 2 | 4 | 5 |
| Adolescents’ fear that parents will be notified about sexual health behaviors | 1 | 2 | 4 | 5 |
| Insufficient knowledge regarding pregnancy risk among adolescents identifying as lesbian, gay, bisexual, transgender, or questioning their sexual orientation | 1 | 2 | 4 | 5 |
| Lack of sufficient time to personally provide care | 1 | 2 | 4 | 5 |
| Insufficient knowledge about where to refer adolescents for reproductive health services | 1 | 2 | 4 | 5 |
| Lack of ancillary staff experience in providing confidential services | 1 | 2 | 4 | 5 |
| Resistance from ancillary staff to provide reproductive health services | 1 | 2 | 4 | 5 |
| Resistance from administration to provide reproductive health services in my practice setting | 1 | 2 | 4 | 5 |

What is your best estimate of the percentage of adolescent patients (aged ³ 14 years) in your practice who have a health condition (e.g., diabetes mellitus, depression, obesity) that could complicate a pregnancy or cause harm to a developing fetus?

<1%

2-10%

11-49%

50-75%

>76%

**Which of the following do you consider when providing reproductive care to an adolescent in your practice?**

1. Informal guidelines in my clinical setting as determined by administrators or practiced by my peers
2. Published guidelines from national medical organizations such as the American Academy of Pediatrics
3. My own review of the evidence-based literature
4. My personal clinical experience
5. Other:__________________________________

How interested would you be in more training and education for any of the following? (Options: Very interested, somewhat interested, not very interested, not at all interested)

1. Taking a sexual history

2. Adolescent Consent and Confidentiality

3. Contraceptive options and management

4. STI/HIV testing for adolescents

5. Other: please list

How likely would you be to increase provision of these services if you had access to increased training/information?

1. Extremely unlikely

2. Unlikely

3. Neutral

4. Likely

5. Extremely likely
